# High systolic blood pressure and stroke: evidence from the NHANES 1999–2023 and global burden of disease 2021

**DOI:** 10.3389/ebm.2026.10868

**Published:** 2026-06-08

**Authors:** LuYi Tang, BoWen Yang, PeiWen Li, YingQi Chen, Yi Liu, Ting Wang, Xiaohan Ye

**Affiliations:** Dongguan Hospital of Guangzhou University of Chinese Medicine, Dongguan, Guangdong, China

**Keywords:** EHANES, global burden of disease (GBD), high systolic blood pressure, stroke, stroke prevention and management

## Abstract

High systolic blood pressure (HSBP) is a major modifiable risk factor for stroke, but trends in disease burden and causal associations related to HSBP in the United States require further investigation using multidimensional approaches. This study aims to elucidate this relationship by utilizing data from the Global Burden of Disease (GBD) database, the National Health and Nutrition Examination Survey (NHANES). This study integrated data from the GBD 2021 database and the NHANES. The GBD data provided macro-level estimates of stroke-related mortality and disability-adjusted life years (DALYs) attributable to various risk factors within the United States. By employing multivariable logistic regression models on individual-level NHANES data, the study assessed the association between HSBP history and stroke risk after adjusting for multiple confounding factors. GBD analysis revealed HSBP as the leading risk factor for U.S. stroke burden in 2021, with an increasing attributable burden since 2010, particularly among the elderly and women. NHANES analysis showed that HSBP significantly increased the risk of stroke (fully adjusted OR = 1.33, 95% CI: 1.17–1.51). Elevated SBP was additionally associated with increased all-cause mortality risk in stroke survivors (HR = 1.01). A novel U-shaped relationship emerged: stroke risk decreased below an SBP of 100 mmHg but increased sharply above this threshold. HSBP is the core driver and modifiable risk factor behind the persistently increasing stroke burden in the United States. The findings of this study highlight the critical importance of HSBP in stroke prevention and management.

## Impact statement

Multidimensional Data Integration: We combine macro-level population burden data (GBD) and individual-level epidemiological data (NHANES), enabling a comprehensive assessment of HSBP’s role in stroke. Novel Epidemiological Insights: Our analysis revealed a Ushaped relationship between systolic blood pressure and stroke risk, identifying a threshold (<130 mmHg) below which stroke risk declines but above which it rises sharply. This nuanced finding contributes to understanding blood pressure management in stroke prevention. Updated and Longitudinal Data Analysis: Using the most recent and extensive datasets spanning over two decades allows us to characterize temporal trends in stroke burden attributable to HSBP, particularly highlighting the rising impact among elderly individuals and women.

## Introduction

Globally, stroke is the second leading cause of death after ischemic heart disease, with approximately 795,000 stroke events occurring annually in the United States, resulting in substantial economic burden, with estimated annual costs of $34 billion [1, 2] for health care services, medications, and work absenteeism. Established cerebrovascular risk factors, including hypertension, hyperlipidemia, atrial fibrillation, diabetes mellitus, smoking, and physical inactivity, significantly contribute to stroke risk. Given the significant impact stroke has on the lives and health of a large proportion of the population, there is an urgent need for timely and effective preventive interventions.

High systolic blood pressure (HSBP) is considered a major risk factor for stroke mortality [3, 4]. At the same time, it is a major risk factor for most cardiovascular diseases, including stroke, and is an even more important predictor than diastolic blood pressure, mean arterial pressure, and pulse pressure. Previous reports indicate that up to 60% of global stroke-related mortality may be associated with poorly controlled high systolic blood pressure, and blood pressure management has been proven to be the most effective intervention for stroke prevention [5].

Despite the heavy burden on the United States healthcare system, there are significant knowledge gaps regarding morbidity and mortality among people of different ages, genders, and economic levels [6]. Although the Global Burden of Disease (GBD) study provides extensive data on stroke risk factors in the United States, it lacks individual-level data on HSBP and stroke [7]. These models often rely on assumptions about the prevalence of high systolic blood pressure or simplistic ecological adjustments, which poses a significant potential for confusion [8, 9]. Therefore, this study aims to provide a more comprehensive assessment of the association between HSBP and stroke risk in the U.S. by integrating data from the GBD database and National Health and Nutrition Examination Survey (NHANES).

In addition, the study aims to identify potential differences in stroke prognosis, including differences in disease burden across socioeconomic groups, ethnicities, and geographic regions. Through this comprehensive analysis, this study is expected to provide valuable insights into reducing the burden of stroke due to HSBP in the United States. The results of this study will provide a scientific basis for strengthening HSBP interventions and early screening strategies, thereby improving the prognosis of stroke in high-risk groups.

## Methods

### Design

This study adopted a complementary two-level design by integrating GBD 2021 and NHANES data. The GBD database was used to quantify the population-level burden of stroke attributable to HSBP in the United States, including temporal and demographic patterns, whereas NHANES was used to assess the individual-level association between HSBP and stroke in a nationally representative sample. This combined design allowed us to examine both the public health significance and the individual-level relationship of HSBP with stroke.

### Data sources and participants

#### GBD database

GBD 2021 is a comprehensive database that includes anonymized data encompassing 204 countries and territories, 7 regions, and 5 Socio-Demographic Index (SDI) quintiles, covering 371 diseases, 88 risk factors, and various types of injuries [4]. The data are publicly accessible through the Global Health Data Exchange (GHDx) (https://vizhub.healthdata.org/gbd-results/), an online platform developed and maintained by the Institute for Health Metrics and Evaluation (IHME) at the University of Washington [10]. Research involving GBD 2021 data was reviewed by the University of Washington’s Institutional Review Board (IRB), which approved a waiver of the informed consent requirement [11]. This study strictly adhered to the GATHER (Guidelines for Accurate and Transparent Health Estimates Reporting) standards to ensure the precision and transparency of health assessment findings [12].

We applied a Bayesian age–period–cohort (BAPC) model fitted with integrated nested Laplace approximations (INLA) to generate predictive distributions of age-standardized mortality and DALY rates to 2045. BAPC explicitly estimates age, period, and cohort effects, and has been demonstrated as an effective approach for forecasting disease rates in large demographic datasets [13].

In the GBD database, the age-standardized mortality rate (ASDR) indicates the number of deaths per 100,000 people by age group. Age-standardized disability-adjusted life years (ASDAR) combine the years of life lost due to premature death and the years lived with disability per 100,000 population after age standardization. The study collected data on stroke and its subtypes attributable to HSBP, including mortality, disability-adjusted life-years (DALYs), age-standardized mortality rate (ASDR), and corresponding 95% uncertainty intervals (UIs). All data were stratified by age, sex, and other demographic variables.

#### NHANES database

NHANES is a stratified, multistage study program conducted by the National Center for Health Statistics (NCHS). It is designed to assess the health and nutritional status of the U.S. population by collecting nationally representative data. It has been widely used to investigate chronic diseases, nutrition, environmental exposures, and the relationships between health and behavior [14]. NHANES data collection and analysis procedures have been previously reported [15]. NHANES is conducted by the Centers for Disease Control and Prevention’s National Center for Health Statistics. Written informed consent was obtained from all participants, and the protocol was approved by the National Center for Health Statistics Institutional Review Board [16]. Representative survey subjects were selected by the method of stratified multi-stage probability sampling. Demographic data, questionnaire data, and dietary data were obtained, including demographics (age, gender, education, race), medical history, diet, and lifestyle habits (smoking, alcohol consumption, exercise). Detailed methods are available on the NHANES website^1^.

**FIGURE 1:**
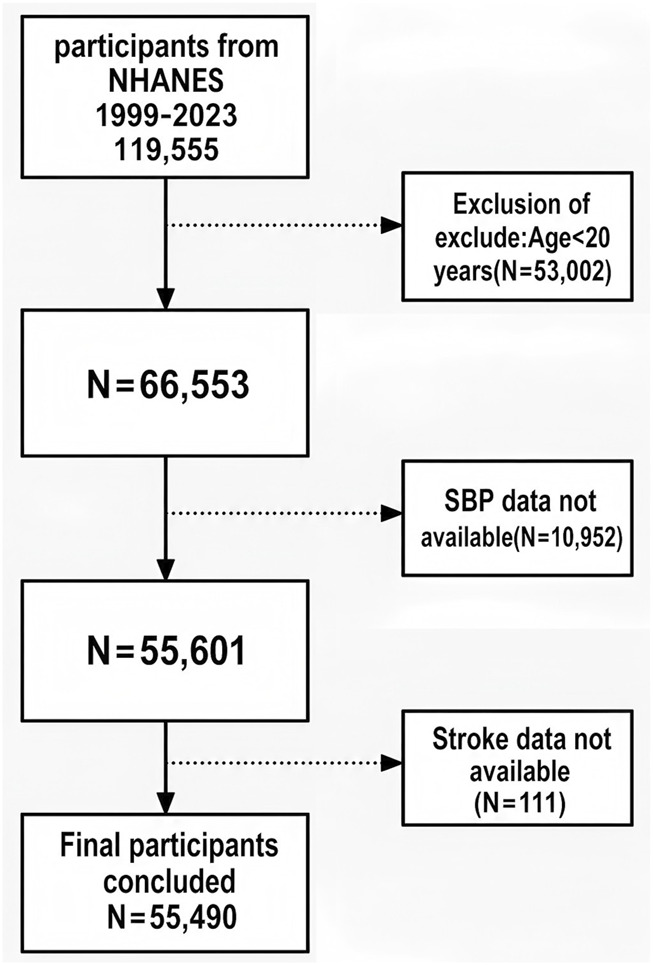
The flow chart of selection of included studies.

This study analyzed data from 12 NHANES cycles (1999–2023). As shown in Figure 1, we initially included 119,555 participants from the NHANES database. This study focused on adults; thus, participants younger than 20 years were excluded. The definition of HSBP (SBP ≥130 mmHg) was based on the 2017 AHA/ACC adult guideline, whereas blood pressure classification in children and adolescents relies on age-, sex-, and height-specific criteria. Moreover, the low occurrence of stroke in individuals younger than 20 years in NHANES may limit the stability and interpretability of age-stratified analyses. After excluding participants aged <20 years (n = 53,002), those with fewer than 3 systolic blood pressure measurements (n = 10,952), and cases with missing stroke information (n = 111), we ultimately included 55,490 eligible participants in our final analysis.

### Measures

#### Primary outcome

The primary outcome variable was stroke. The Medical Status Questionnaire was used to obtain a self-reported stroke diagnosis. Respondents were asked, “Have doctors or other health professionals ever told you about a stroke?” Based on their responses to this self-reported question, participants were divided into either the “stroke” or “non-stroke” groups. Previous studies have validated the use of self-reported stroke [17, 18].

#### Key variables

The primary exposure is the presence or absence of HSBP. In NHANES, all blood pressure measurements were taken by a certified examiner using a sphygmomanometer after the participant sat still for 5 min. According to the 2017 American Heart Association/American College of Cardiology (AHA/ACC) guidelines [19], individuals with SBP ≥130 mmHg were classified as having HSBP. To minimize random measurement error, we only included participants who had ≥3 SBP measurements, with the final SBP value calculated as the mean of three separate blood pressure readings.

#### Covariates

In the NHANES database, participant age was calculated from the date of interview to the date of birth, while gender was coded by NHANES personnel as either male or female following standardized protocols. Race is assessed through two questions: (1) “Do you identify as Hispanic, Mexican, or non-Hispanic?” (2) “What race do you consider yourself?” Based on responses to these questions, NHANES personnel categorized participants into five mutually exclusive racial/ethnic groups: Mexican American, Other Hispanic, Non-Hispanic White, Non-Hispanic Black, and Other Race. Education levels are grouped by the following question: “What is the highest grade or level of school you completed or the highest degree you earned?” According to the answer to this question, education is divided into three groups: below high school, high school, and above high school. Marital status is divided into married/partnered, divorced/widowed/separated, and never married. The PIR represents the ratio of a family’s self-reported income to the appropriate poverty threshold. Participants were stratified into three groups based on PIR values: <1.3, 1.3–3.5, and ≥3.5. Body mass index (BMI) was calculated as weight in kilograms divided by the square of height in meters (kg/m^2^). Participants were categorized into three BMI groups: <25 kg/m^2^ (underweight/normal weight), 25 to <30 kg/m^2^ (overweight), and ≥30 kg/m^2^ (obese). Alcohol consumption, exercise, and smoking are divided into two categories: yes or no. Diabetes was defined as meeting any one of the following criteria: a documented history of diabetes, current use of insulin, use of oral hypoglycemic medications, glycated hemoglobin (HbA1c) ≥6.5%, fasting plasma glucose ≥126 mg/dL (7.0 mmol/L), or 2-h postprandial glucose ≥200 mg/dL (11.1 mmol/L). The diagnosis of hyperlipidemia was based on the National Cholesterol Education Program Adult Treatment Panel III (NCEP-ATP III) guidelines (2002). Participants were identified as having hyperlipidemia if they met any of the following criteria: (1) TC ≥ 200 mg/dL, (2) TG ≥ 150 mg/dL, (3) LDL-C ≥ 130 mg/dL, or (4) HDL-C ≤ 40 mg/dL for males or ≤50 mg/dL for females. In addition, participants taking lipid-lowering drugs were also considered to have hyperlipidemia.

Participants were categorized into either low or high physical activity groups based on whether they met the National Physical Activity Guidelines (low physical activity: <500 MET-min/week; high physical activity: ≥500 MET-min/week) [20]. To ensure data quality, a comprehensive evaluation of missing data was conducted. For variables with a missing data rate of more than 10%, a multiple imputation approach was used.

This manuscript has been reported in line with the STROCSS criteria [21]. We utilized de-identified data from public databases, including the GBD and the NHANES. Ethical approval was obtained in the original surveys and was not required for this study.

### Statistical analysis

This study utilized GBD 2021 data from the GBD database to analyze risk factors contributing to the stroke burden in the United States. We assessed each risk factor’s attributable deaths, attributable DALYs, and age-standardized attributable DALY rates. NHANES data were utilized to analyze the association between HSBP and stroke incidence. Continuous variables were expressed as mean ± standard deviation, while categorical variables were presented as case numbers (percentages). Between-group comparisons were conducted using t-tests, Mann-Whitney U tests, or χ^2^ tests. Multivariable logistic regression models were applied to examine the association between HSBP and stroke incidence.

Three multivariate logistic regression models were constructed to evaluate the linear relationship between HSBP and stroke: Model 1, unadjusted; Model 2, adjusted for age, gender, and race; and Model 3, further adjusted for education, marital status, smoking history, alcohol consumption, physical activity, diabetes, hyperlipidemia, and PIR based on Model 2. The results were presented as odds ratios (ORs) with corresponding 95% confidence intervals (CIs). Cox proportional hazards regression analysis was performed to evaluate the association between SBP and all-cause mortality among stroke survivors. Covariates included in the three models were consistent with those used in the multivariable regression analyses. Sensitivity analyses were additionally conducted treating systolic blood pressure as a continuous variable and stroke occurrence as the outcome, to assess the impact of each unit increase in systolic blood pressure on stroke incidence. Subgroup stratification and interaction tests were performed to investigate the association between HSBP and stroke across various demographic groups. The results of subgroup analyses were illustrated using forest plots. To account for NHANES’s complex probability cluster sampling design, survey weights were incorporated in all statistical analyses. For covariates with less than 15% missing data, quantitative variables were imputed using the Predictive Mean Matching (PMM) method, while categorical variables were imputed via logistic regression imputation.

## Results

### HSBP is a major risk factor contributing to the stroke burden in the United States

**FIGURE 2:**
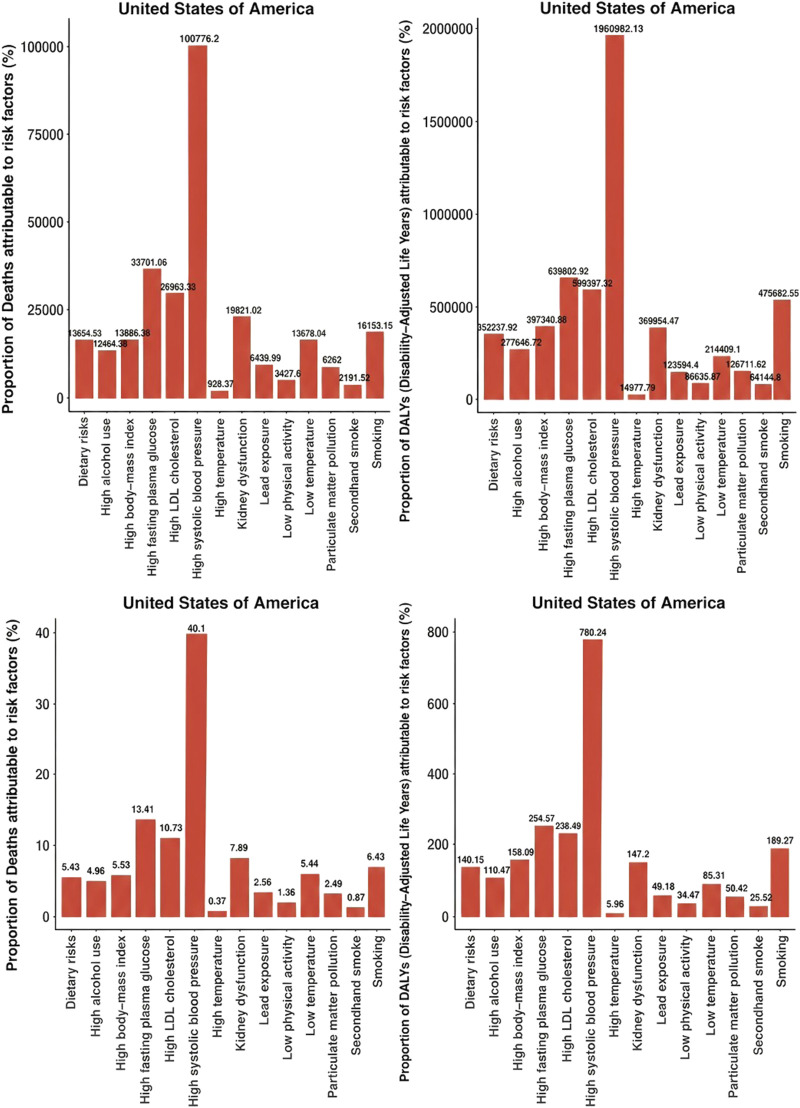
2021 US stroke burden by risk factors.

Figure 2 summarizes the stroke burden attributable to various risk factors in the United States in 2021. In 2021, HSBP was the leading contributor among stroke risk factors, significantly exceeding all others. This dominance was consistently observed across all metrics: deaths (100,776.20; 95% uncertainty interval [UI]: 70,492.27–129,333.71), DALYs (1,960,982.13; 95% UI: 1,421,828.68–2,483,402.75), ASDR (40.10; 95% UI: 28.05–51.46), and ASDAR (780.24; 95% UI: 565.72–988.10).

**FIGURE 3:**
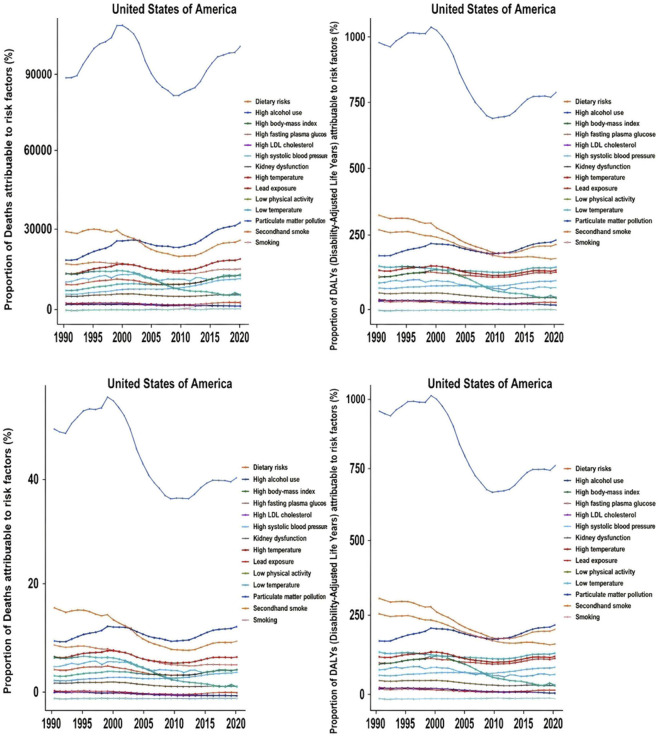
US time-stratified stroke burden from HSB.

Figure 3 presents a comprehensive temporal analysis of the stroke burden attributable to HSBP based on GBD study data. Results indicate that from 1990 to 1999, the stroke burden attributable to HSBP exhibited an overall increasing trend, followed by a consistent decline from 1999 to 2010. Subsequently, the trend reversed once more, rising after 2010. This pattern was consistently observed across all four metrics: deaths, DALYs, ASDR, and ASDAR.

**FIGURE 4:**
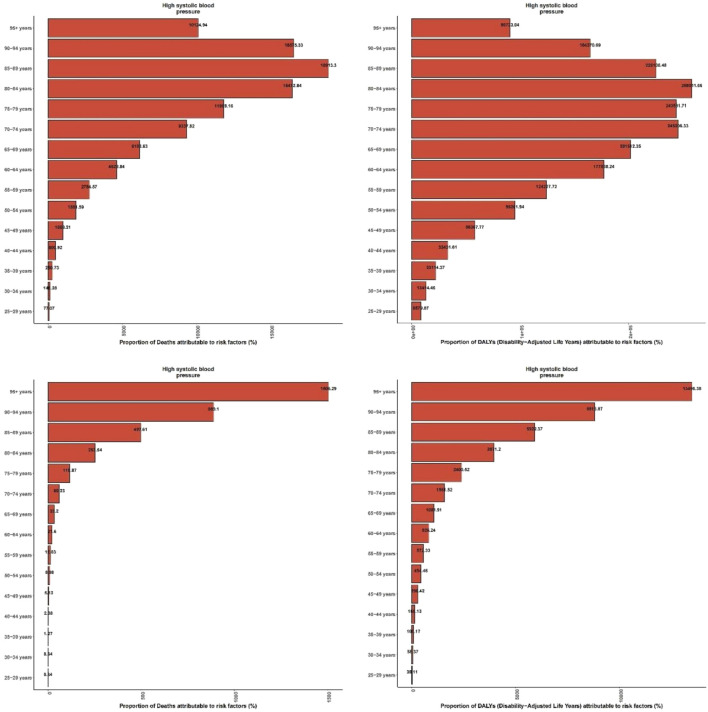
US age-stratified stroke burden from HSBP.

Figure 4 presents an age-stratified analysis of the stroke burden attributable to HSBP in the United States. Results show that stroke deaths attributed to HSBP peaked in the 85–89 age group, with DALYs predominantly distributed among those aged 80–84 years; both ASDR and ASDAR reached their highest values in the population aged ≥95 years.

**FIGURE 5:**
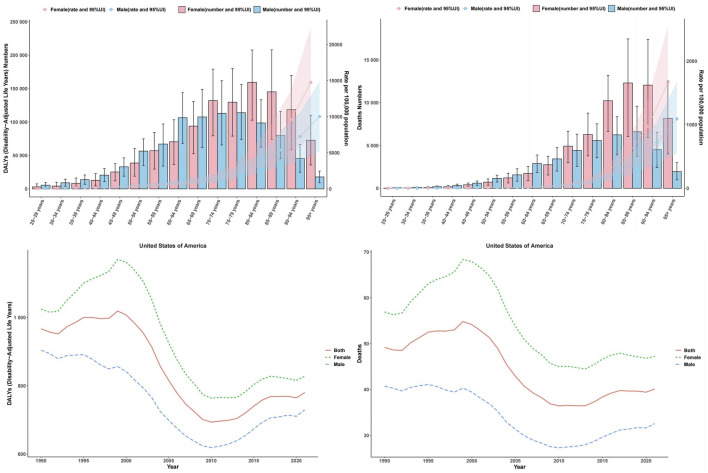
Gender-stratified analysis.

Gender-stratified analysis revealed male predominance in SBP-attributable stroke mortality, DALYs, and ASDAR among individuals aged 25–69 years in 2021, whereas female predominance was observed after age 70. Although ASDR was higher in males aged 25–79 years, females exhibited higher ASDR after age 80. From 1990 to 2021, females consistently exhibited higher ASDR and ASDAR than males (Figure 5).

**FIGURE 6:**
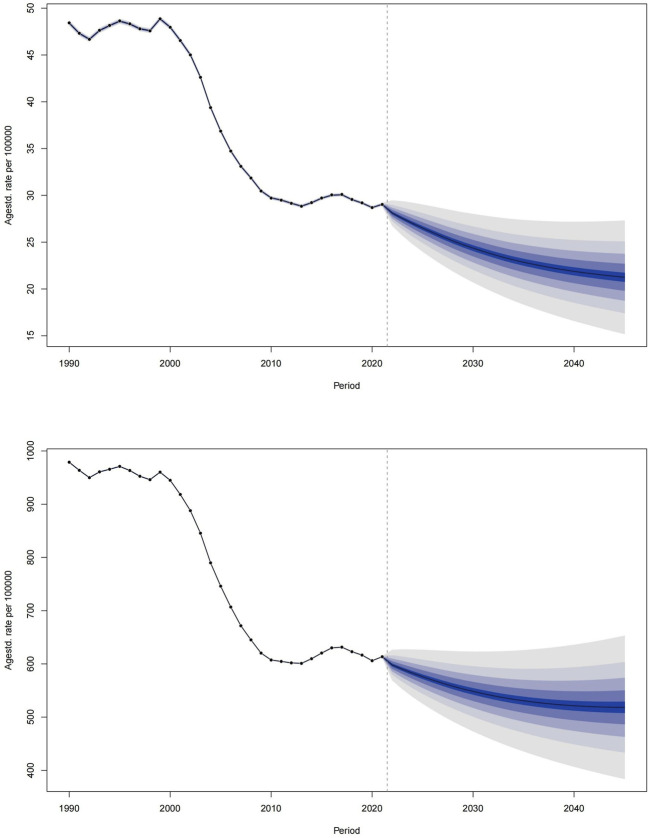
Predictive model.

In 2021, the ASDR for SBP-attributable stroke in the United States was 29.04 (95% UI: 28.86–29.22), with projections indicating a decline to 21.25 (95% UI: 14.05–28.45) by 2045. Similarly, the ASDAR was 613.48 (95% UI: 605.13–606.89) in 2021, with projections estimating a decrease to 518.46 (95% UI: 358.43–678.48) by 2045. The analysis predicts a consistent downward trend for both ASDR and ASDAR from 2021 through 2045 (Figure 6).

### Characteristics of NHANES study participants

This study included data from 119,555 participants. After excluding subjects younger than 20 years or lacking mean SBP or necessary stroke data, a total of 55,490 participants were finally enrolled (see flow chart). The study population comprised 53,363 non-stroke individuals and 2,127 stroke patients. Table 1 presents the detailed demographic characteristics of the study population stratified by stroke status. Significant differences were observed between stroke and non-stroke subgroups in variables including race, education level, marital status, smoking history, alcohol consumption, METs, diabetes status, hyperlipidemia, mean SBP, age, and PIR. However, no statistically significant difference was found in sex distribution between the two groups. Compared to non-stroke individuals, stroke patients were significantly more likely to be older, of non-Hispanic White race, less educated, current smokers, alcohol consumers, diabetic, hyperlipidemic, and to have a lower PIR (all P < 0.05).

**TABLE 1:** NHANES 1999-2023 participant baseline traits.

Variable	Total (n = 55,490)	0 (n = 53,363)	1 (n = 2,127)	Statistic	*P*
Sex, n (%)	​	​	​	χ^2^ = 5.29	0.060
Male	26,849 (48.57)	25,767 (48.65)	1,082 (45.68)	​	​
Female	28,641 (51.43)	27,596 (51.35)	1,045 (54.32)	​	​
Age, n (%)	​	​	​	χ^2^ = 1,352.06	**<0.001**
20–59	36,241 (74.68)	35,682 (75.83)	559 (34.47)	​	​
≥60	19,249 (25.32)	17,681 (24.17)	1,568 (65.53)	​	​
SBP, n (%)	​	​	​	χ^2^ = 404.94	**<0.001**
<130	37,698 (73.04)	36,704 (73.68)	994 (50.59)	​	​
≥130	17,792 (26.96)	16,659 (26.32)	1,133 (49.41)	​	​
Race, n (%)	​	​	​	χ^2^ = 53.34	**<0.001**
Mexican American	8,730 (7.94)	8,523 (8.04)	207 (4.35)	​	​
Other hispanic	4,835 (6.05)	4,697 (6.09)	138 (4.57)	​	​
Non-hispanic white	25,162 (67.69)	24,098 (67.65)	1,064 (68.80)	​	​
Non-hispanic black	11,348 (10.95)	10,787 (10.84)	561 (14.82)	​	​
Other race	5,415 (7.37)	5,258 (7.37)	157 (7.47)	​	​
Education level, n (%)	​	​	​	χ^2^ = 238.86	**<0.001**
Less than high school	13,591 (15.78)	12,848 (15.48)	743 (26.43)	​	​
High school	12,788 (24.19)	12,205 (23.98)	583 (31.79)	​	​
More than high school	29,111 (60.02)	28,310 (60.54)	801 (41.78)	​	​
Martial status, n (%)	​	​	​	χ^2^ = 232.80	**<0.001**
Married/living with partner	33,083 (63.84)	31,999 (64.05)	1,084 (56.69)	​	​
Widowed/divorced/separated	14,997 (22.62)	14,073 (22.20)	924 (37.38)	​	​
Never married	7,410 (13.54)	7,291 (13.76)	119 (5.93)	​	​
Smoke, n (%)	​	​	​	χ^2^ = 113.07	**<0.001**
No	30,481 (54.53)	29,636 (54.91)	845 (41.22)	​	​
Yes	25,009 (45.47)	23,727 (45.09)	1,282 (58.78)	​	​
Drinking, n (%)	​	​	​	χ^2^ = 56.99	**<0.001**
No	16,549 (25.40)	15,813 (25.17)	736 (33.67)	​	​
Yes	38,941 (74.60)	37,550 (74.83)	1,391 (66.33)	​	​
Physical activity, n (%)	​	​	​	χ^2^ = 284.52	**<0.001**
Low physical activity	21,248 (34.58)	20,023 (34.00)	1,225 (54.75)	​	​
High physical activity	34,242 (65.42)	33,340 (66.00)	902 (45.25)	​	​
Diabetes, n (%)	​	​	​	χ^2^ = 842.34	**<0.001**
No	45,700 (86.79)	44,446 (87.49)	1,254 (62.07)	​	​
Yes	9,790 (13.21)	8,917 (12.51)	873 (37.93)	​	​
Hyperlipidemia, n (%)	​	​	​	χ^2^ = 132.24	**<0.001**
No	17,902 (32.48)	17,452 (32.87)	450 (18.94)	​	​
Yes	37,588 (67.52)	35,911 (67.13)	1,677 (81.06)	​	​
BMXBMI, n (%)	​	​	​	χ^2^ = 63.07	**<0.001**
<25	16,125 (30.42)	15,613 (30.61)	512 (23.60)	​	​
25–30	18,649 (33.31)	17,970 (33.37)	679 (31.00)	​	​
≥30	20,716 (36.27)	19,780 (36.01)	936 (45.41)	​	​
PIR, n (%)	​	​	​	χ^2^ = 205.25	**<0.001**
<1.3	16,614 (21.29)	15,758 (20.96)	856 (32.58)	​	​
1.3–3.5	21,049 (36.00)	20,187 (35.86)	862 (41.07)	​	​
>=3.5	17,827 (42.71)	17,418 (43.18)	409 (26.34)	​	​

SE: standard error.

t: t-test, χ^2^: Chi-square test.

### Association between HSBP and stroke

All models consistently indicated that HSBP was associated with an increased incidence of stroke (Table 2). Specifically, Model 1 showed an odds ratio (OR) of 2.73 (95% confidence interval [CI]: 2.45–3.05); Model 2, OR = 1.52 (95% CI: 1.33–1.72); and Model 3, OR = 1.33 (95% CI: 1.17–1.51), indicating that individuals with HSBP had a 1.33-fold higher risk of stroke compared to those with normal blood pressure.

**TABLE 2:** The association between HSBP and stroke.

Variables	Model1		Model2		Model3	
	OR (95%CI)	*P*	OR (95%CI)	*P*	OR (95%CI)	*P*
SBP						
<130	1.00 (reference)	​	1.00 (reference)	​	1.00 (reference)	​
≥130	2.73 (2.45–3.05)	**<0.001**	1.52 (1.33–1.72)	**<0.001**	1.33 (1.17–1.51)	**<0.001**
P for trend	**<0.001**	​	**<0.001**	​	**<0.001**	​

OR: odds ratio, CI: confidence interval.

Model1: Crude.

Model2: Adjust: Adjusted for sex, age and race.

Model3: Adjusted for sex, age, race, education, marital status, smoking history, alcohol consumption, physical activity, diabetes, hyperlipidemia, and PIR.

### Association of HSBP and all-cause mortality in stroke survivors

Among 1,711 stroke survivors, 743 all-cause deaths occurred during the follow-up period. The unadjusted regression analysis (Table 3) indicated that HSBP was associated with an increased risk of all-cause mortality among stroke survivors (hazard ratio [HR] = 1.01; 95% CI: 1.01–1.02), suggesting a positive correlation between HSBP and mortality risk. This association remained statistically significant in the fully adjusted model (Model 3), with an HR of 1.01 (95% CI: 1.01–1.01). These results consistently demonstrate that elevated SBP significantly increases all-cause mortality risk among stroke survivors (Table 3).

**TABLE 3:** Association between SBP and all-cause mortality in stroke survivors.

Variables	Model1		Model2		Model3	
	HR (95%CI)	*P*	HR (95%CI)	*P*	HR (95%CI)	*P*
Systolic blood pressure	1.01 (1.01–1.02)	**<0.001**	1.01 (1.01–1.01)	**0.003**	1.01 (1.01–1.01)	**0.010**

HR: hazard ratio, CI: confidence interval.

Model1: Crude.

Model2: Adjusted for sex, age and race.

Model3: Adjusted for sex, age, race, education, marital status, smoking history, alcohol consumption, physical activity, diabetes, hyperlipidemia, and PIR.

### Subgroup analysis

**FIGURE 7:**
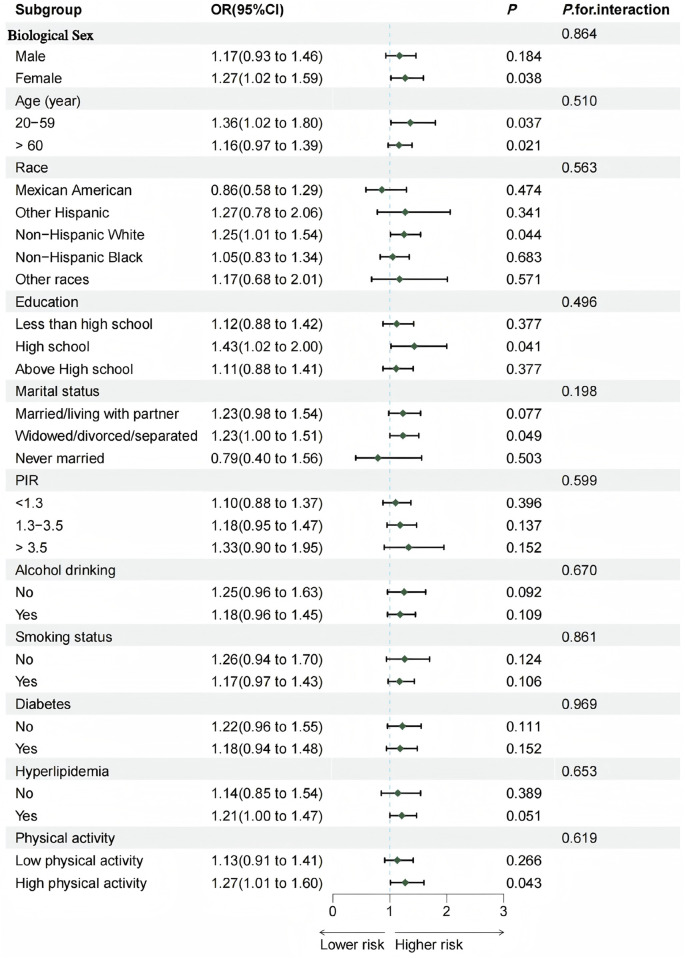
Subgroup analysis.

To assess the consistency of the association between HSBP and stroke across different populations, subgroup analyses and interaction tests stratified by gender, race, age, education level, marital status, alcohol consumption, physical activity, smoking history, diabetes mellitus, hyperlipidemia, mean SBP, and poverty-income ratio (PIR) were performed. As shown in Figure 7, no covariate significantly modified the association between HSBP and stroke.

### Sensitivity analysis

In the sensitivity analysis, systolic blood pressure was treated as a continuous variable, and stroke occurrence was used as the outcome; multivariable logistic regression analysis was conducted to evaluate this relationship. In the fully adjusted model, HSBP remained significantly associated with increased stroke incidence (P < 0.05), with an adjusted OR of 1.01 (95% CI: 1.01–1.01). Weighted logistic regression models employed in the sensitivity analyses confirmed the robustness of these findings, demonstrating consistent associations between HSBP and stroke prevalence in alignment with primary analysis results (Table 4).

**TABLE 4:** Sensitivity analysis.

Variables	Model1		Model2		Model3	
	OR (95%CI)	*P*	OR (95%CI)	*P*	OR (95%CI)	*P*
Systolic blood pressure	1.03 (1.02–1.03)	**<0.001**	1.01 (1.01–1.01)	**<0.001**	1.01 (1.01–1.01)	**<0.001**

OR: odds ratio, CI: confidence interval.

Model1: Crude.

Model2: Adjusted for sex, age and race.

Model3: Adjusted for sex, age, race, education, marital status, smoking history, alcohol consumption, physical activity, diabetes, hyperlipidemia, and PIR.

### Nonlinear relationship between HSBP and stroke

**FIGURE 8:**
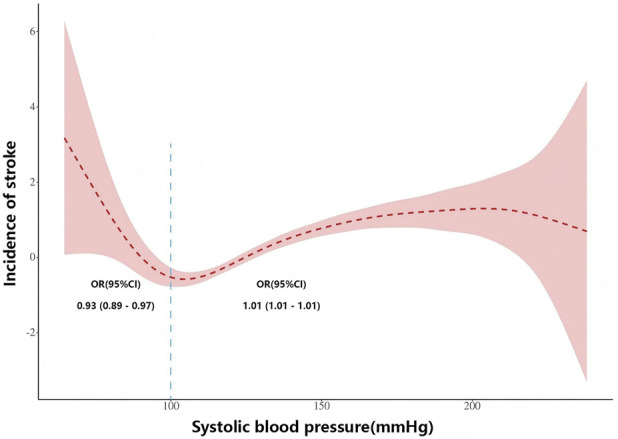
Nonlinear relationship between HSBP and stroke.

Figure 8 illusrates the relationship between HSBP and stroke prevalence based on NHANES data. After adjustment for sex, race, education level, marital status, smoking history, alcohol consumption, METs, diabetes status, hyperlipidemia, BMI, age, and PIR, smoothed curve fitting revealed a U-shaped association between SBP and stroke prevalence. A piecewise regression model identified 100 mmHg as the critical threshold for SBP. Results demonstrated that within the lower SBP range (<100 mmHg), stroke risk decreased as blood pressure increased; conversely, above this threshold (>100 mmHg), higher SBP levels were associated with progressively increased stroke risk.

## Discussion

A major strength of this study is the complementary use of GBD and NHANES data. The GBD analysis established the population-level context by showing that HSBP was the leading contributor to stroke burden in the United States, whereas the NHANES analysis extended these findings by demonstrating that HSBP was independently associated with stroke in a nationally representative sample. Together, these results provide both epidemiological context and individual-level support for the role of HSBP as a major modifiable risk factor for stroke. Our findings demonstrate that HSBP remains the predominant risk factor for stroke-related deaths and DALYs in the U.S. Notably, the attributable burden has exhibited a consistent year-over-year increase since 2010. In the fully adjusted multivariable logistic regression model, individuals with HSBP demonstrated a 1.33-fold increased risk of stroke compared to those with normal blood pressure levels. Furthermore, each 1 mmHg increase in SBP was associated with approximately a 1% increase in the risk of stroke incidence (OR = 1.01, 95% CI: 1.01–1.01). These findings not only quantify the stroke disease burden attributable to SBP at the U.S. population level but also confirm HSBP as a key modifiable risk factor based on large-scale population data, thereby providing critical evidence for developing and optimizing targeted stroke prevention strategies, particularly in the U.S. where the burden continues to rise.

Our study confirms that HSBP is a key independent risk factor for stroke, a finding that is highly consistent with the extensive epidemiological evidence accumulated to date. A previous meta-analysis of the relationship between SBP and cardiovascular disease risk demonstrated that elevated SBP levels are the leading global cause of total mortality and a major contributor to long-term disability [22], with a greater predictive value than diastolic, mean arterial, and pulse blood pressure [23, 24]. The study data demonstrated that each 20 mmHg increase in SBP was associated with significantly increased risks across stroke subtypes: a 35% higher risk of ischemic stroke (95% CI: 1.28–1.42), 44% increased risk of intracerebral hemorrhage (95% CI: 1.32–1.58), and 43% greater risk of subarachnoid hemorrhage (95% CI: 1.25–1.63) [25]. Moreover, this level of blood pressure elevation more than doubled the risk of all-cause mortality [26].

Several underlying mechanisms may account for the observed positive association between HSBP and stroke. First, hypertension profoundly impacts the structure of cerebral blood vessels. Mechanical, neural, and humoral factors collectively contribute to structural alterations in the vascular wall. Under chronic high-pressure conditions, smooth muscle cells undergo hypertrophy, hyperplasia, and disorganized proliferation, which encroaches upon the arterial lumen. This pathological remodeling results in vascular narrowing, wall thickening, and arterial stiffening [27–29], thereby promoting atherosclerotic plaque formation and eventually leading to arterial occlusion and ischemic injury. Secondly, in patients with HSBP, decreased cerebral blood flow may precede cerebrovascular symptoms or white matter lesions. This reduction may be secondary to endothelial dysfunction, leading to increased vascular tone [30]. Furthermore, endothelial dysfunction impairs autoregulatory function [31], necessitating higher perfusion pressure to maintain normal cerebral blood flow. Concurrently, HSBP directly induces oxidative stress in the cerebrovasculature [32]. Such persistent oxidative stress depletes antioxidant molecules and inactivates antioxidant enzymes, compromising the endogenous antioxidant defense system and exacerbating both structural and functional vascular damage [33]. Moreover, inflammation constitutes a key pathological mechanism in vascular injury [34]. Inflammatory biomarkers have been shown to predict the risk of primary ischemic stroke [35]. Individuals predisposed to stroke consistently exhibit elevated levels of inflammatory markers—including C-reactive protein (CRP), interleukin-6 (IL-6), leukocyte elastase, lipoprotein(a), intercellular adhesion molecule-1 (ICAM-1), and E-selectin—compared to stroke-free individuals [36]. Finally, baroreflex sensitivity (BRS) is a key indicator of arterial baroreceptor function and plays a critical role in both the pathogenesis and prognosis of stroke [37]. After ischemic or hemorrhagic stroke, baroreflex dysfunction and blood pressure variability may significantly alter cerebral perfusion and exacerbate perihematoma edema [37, 38]. Therefore, in the absence of intervention, oxidative stress, inflammation, and baroreflex dysfunction may create a vicious cycle that ultimately culminates in stroke.

This study comprehensively evaluated the association between HSBP and stroke risk burden in the U.S. by integrating two complementary approaches: large-scale, nationally representative NHANES data and GBD estimates. This study highlights the ongoing increase in the burden of HSBP-related stroke in the U.S. and confirms HSBP as a key modifiable risk factor for this disease. These findings provide a strong scientific basis for the urgent reinforcement of comprehensive blood pressure control measures in the U.S. In light of this rising and preventable disease burden, immediate, multidimensional, and targeted interventions are imperative. The restricted cubic spline analysis further suggests that the relationship between SBP and stroke is nonlinear, with a threshold near 100 mmHg, indicating that both elevated SBP and excessively low SBP in certain contexts may warrant clinical attention. These findings have important policy implications, as they support greater funding for blood pressure screening, early intervention, and long-term stroke prevention programs. Although based on U.S. data, the present results may also be informative globally, given that HSBP remains a leading modifiable risk factor for stroke worldwide and may represent a practical target for prevention across diverse healthcare settings.

However, this study has several limitations. Stroke inclusion criteria relied on self-reported stroke history, and the stroke subtypes were not identified, preventing further assessment of associations between HSBP and specific stroke subtypes. Furthermore, the findings are primarily applicable to individuals aged 20 years and above in the United States, limiting the generalizability of results. Future studies should consider more refined age stratification, such as 20–35 years, 36–45 years, and 46–60 years, to better characterize potential age-related heterogeneity in the association between HSBP and stroke. Nevertheless, the established association between HSBP and stroke is robust enough that it is unlikely to be substantially affected by unmeasured confounding factors. Future research should further investigate the long-term effects of HSBP on stroke using longitudinal designs and multicenter stroke registry data.

## Conclusion

In summary, our findings demonstrate that HSBP is a core driver of the persistently increasing stroke burden in the United States and a crucial target for interventions aimed at preventing stroke-related morbidity. Our results hold significant implications for clinical stroke risk management and prevention. We anticipate that future longitudinal studies and multicenter stroke registry data analyses will further validate our conclusions.

## Data Availability

The data sets generated and/or analyzed during the current study are available in the GBD repository (http://ghdx.healthdata.org/gbd-results-tool). The original contributions presented in the study are included in the article/supplementary material. Further inquiries can be directed to the corresponding author.

## Footnotes

1
https://www.cdc.gov/nchs/nhanes/
